# A Quorum-Sensing System That Regulates *Streptococcus pneumoniae* Biofilm Formation and Surface Polysaccharide Production

**DOI:** 10.1128/mSphere.00324-17

**Published:** 2017-09-13

**Authors:** Roger Junges, Gabriela Salvadori, Sudhanshu Shekhar, Heidi A. Åmdal, Jimstan N. Periselneris, Tsute Chen, Jeremy S. Brown, Fernanda C. Petersen

**Affiliations:** a Department of Oral Biology, Faculty of Dentistry, University of Oslo, Oslo, Norway; b Centre for Inflammation and Tissue Repair, Division of Medicine, University College Medical School, Rayne Institute, London, United Kingdom; c Department of Microbiology, The Forsyth Institute, Cambridge, Massachusetts, USA; University of Iowa

**Keywords:** *Streptococcus pneumoniae*, biofilms, cell signaling, mutagenesis, natural transformation systems, polysaccharides, quorum sensing, *rgg*, *shp*, transcriptional regulation

## Abstract

Despite vaccines, *Streptococcus pneumoniae* kills more than a million people yearly. Thus, understanding how pneumococci transition from commensals to pathogens is particularly relevant. Quorum sensing regulates collective behaviors and thus represents a potential driver of commensal-to-pathogen transitions. Rgg/small hydrophobic peptide (SHP) quorum-sensing systems are widespread in streptococci, yet they remain largely uncharacterized in *S. pneumoniae*. Using directional transcriptome sequencing, we show that the *S. pneumoniae* D39 Rgg0939/SHP system induces the transcription of a single gene cluster including *shp* and capsule gene homologs. Capsule size measurements determined by fluorescein isothiocyanate-dextran exclusion allowed assignment of the system to the regulation of surface polysaccharide expression. We found that the SHP pheromone induced exopolysaccharide expression in R36A, an unencapsulated derivative of D39. In the encapsulated parent strain, overexpression of the Rgg system resulted in a mutant with increased capsule size. In line with previous studies showing that capsule expression is inversely associated with biofilm formation, we found that biofilm formed on lung epithelial cells was decreased in the overexpression strain and increased in an *rgg* deletion mutant. Although no significant differences were observed between D39 and the *rgg* deletion mutant in a mouse model of lung infection, in competitive assays, overexpression reduced fitness. This is the first study to reveal a quorum-sensing system in streptococci that regulates exopolysaccharide synthesis from a site distinct from the original capsule locus.

**IMPORTANCE** Quorum sensing regulates bacterial social behaviors by production, secretion, and sensing of pheromones. In this study, we characterized a new quorum-sensing system of the Rgg/SHP class in *S. pneumoniae* D39. The system was found to directly induce the expression of a single gene cluster comprising the gene for the SHP pheromone and genes with putative functions in capsule synthesis. Capsule size, as measured by dextran exclusion, was increased by SHP exposure in R36A, an unencapsulated derivative of D39. In the encapsulated parent strain, overexpression of the gene cluster increased capsule size, supporting the role of Rgg/SHP in the synthesis of surface polysaccharides. Further, we found that biofilm formation on epithelial cells was reduced by overexpression of the system and increased in a mutant with an *rgg* deletion. Placing surface polysaccharide expression under quorum-sensing regulation may enable *S. pneumoniae* to tune interactions with the host and other bacteria in accordance with environmental and cell density conditions.

## Abstract (precis)

Quorum sensing regulates bacterial social behaviors by production, secretion, and sensing of pheromones. In this study, we characterized a new quorum-sensing system of the Rgg/SHP class in *S. pneumoniae* D39. The system was found to directly induce the expression of a single gene cluster comprising the gene for the SHP pheromone and genes with putative functions in capsule synthesis. Capsule size, as measured by dextran exclusion, was increased by SHP exposure in R36A, an unencapsulated derivative of D39. In the encapsulated parent strain, overexpression of the gene cluster increased capsule size, supporting the role of Rgg/SHP in the synthesis of surface polysaccharides. Further, we found that biofilm formation on epithelial cells was reduced by overexpression of the system and increased in a mutant with an *rgg* deletion. Placing surface polysaccharide expression under quorum-sensing regulation may enable *S. pneumoniae* to tune interactions with the host and other bacteria in accordance with environmental and cell density conditions.

## INTRODUCTION

*Streptococcus pneumoniae* is a colonizer of the nasopharynx and a major human pathogen that causes noninvasive and invasive diseases such as otitis media, meningitis, pneumonia, and septicemia (1, 2). The burden of pneumococcal disease is estimated to be over 1 million fatalities per year, and the majority of deaths occur in children under 5 years of age (3). Prior to infection, *S. pneumoniae* is present in the nasopharyngeal tract as a colonizer. Nasopharyngeal colonization by pneumococci is more frequent in children, peaking around the age of 3 years and progressively declining throughout adulthood, to increase again in elderly people (4–6). The transition from an asymptomatic carrier state to dissemination into other parts of the human body is, however, a complex and dynamic process that is not yet entirely understood (7, 8). In some bacteria, collective behaviors controlled by quorum sensing are emerging as important mechanisms behind such transitions, with examples ranging from inhibition effects of virulence and biofilm formation in *Vibrio cholerae* (9) to enhancement of virulence and promotion of biofilms by the Agr system in *Staphylococcus aureus* (10) and by the Fsr system in *Enterococcus faecalis* (11).

A decade ago, a new pheromone system comprising Rgg regulators and small hydrophobic peptides (SHPs) was discovered in *Streptococcus thermophilus* (12). Genome-wide surveys showed later that such systems are present in the majority of streptococci (13, 14). The *shp* and *rgg* genes have a characteristic arrangement, with the gene for the SHP pheromone being located in the vicinity of flanking regions of *rgg*. Recently described Rgg/SHP systems have been shown to regulate biofilm formation in *S. pyogenes* (15) and virulence in *S. agalactiae* (16). Interestingly, the mechanism of transcriptional regulation that each Rgg employs can vary. In *S. pyogenes*, Rgg2 activates transcription, while Rgg3 represses it (15). Remarkably, the two regulators have been demonstrated to interact with both SHP2 and SHP3, and they both compete for binding to the same promoter areas of the genome located immediately upstream of *shp2* and *shp3* (15, 17). By doing so, they control the same function in opposite ways; while Rgg2 promotes biofilm formation, Rgg3 represses it (15). Moreover, the expression of both *shp2* and *shp3* is greatly increased in the presence of the peptides, since their binding to Rgg3 promotes release of the complex from the *shp* promoter sites, allowing for binding of the Rgg2-SHP complex and activation of *shp* gene transcription (17, 18).

A previous large-scale survey reported the presence of potential Rgg/SHP systems in different strains of *S. pneumoniae* (14); one of these candidates (SPD_1745) was recently described as a TprA/PhrA quorum-sensing system that regulates a lantibiotic biosynthesis gene cluster (19). In addition, Bortoni et al. described a stand-alone Rgg-like transcriptional regulator (SPD_1952) in *S. pneumoniae* D39 that is involved in the response to oxidative stress (20). Here we report the characterization of an Rgg/SHP pheromone system in *S. pneumoniae* serotype 2 virulent strain D39. We show that the system regulates a total of 12 genes organized into a single transcript initiated by the *shp* gene. Further, we present evidence that the regulated locus is involved in the production of a thicker polysaccharide capsule and that its overexpression reduces fitness *in vivo* in a mouse model of lung infection and biofilm formation on epithelial lung cells, whereas deletion of the system promotes biofilm formation.

## RESULTS

### Presence of Rgg-like genes in *S. pneumoniae* strain D39.

Potential Rgg/SHP candidates have been screened in *S. pneumoniae* R6 (D39 derivative) and TIGR4 (13, 14). However, since SHPs are not annotated and may be difficult to identify, we performed an additional search of the sequenced *S. pneumoniae* strain D39 for the presence of putative SHP peptides in the proximity of homologous Rgg regulators. Homology searching using the sequences of the characterized Rgg proteins from *S. pyogenes* NZ131—Spy49_0415/Rgg2 and Spy49_0449c/Rgg3—and from *S. agalactiae* NEM316—*gbs1555*/RovS—retrieved four sequences with at least 20% protein identity (SPD_0939, SPD_0999, SPD_1952, SPD_0144). The highest identity was found in SPD_0939, with 53.5, 89.7, and 49.2% identity to Spy49_0415, Spy49_0449c, and *gbs1555*, respectively. We manually searched in the vicinity of these genes for nonannotated open reading frames (ORFs) that could contain *shp* genes. Confirming previous predictions, the only candidate pheromone we found was adjacent to SPD_0939 (13, 14).

A detailed promoter analysis and a map of the Rgg0939/SHP system in D39 are presented in Fig. S1 in the supplemental material. The predicted binding site for Rgg in the *shp* promoter of D39 is similar to those previously described for *shp3* and *shp2* in *S. pyogenes* and *shp1555* in *S. agalactiae* (16, 17). Furthermore, we assessed the presence of this system in other strains of *S. pneumoniae* by using Rgg0939 as a template and found that 12 (36.4%) of the 33 fully sequenced strains of *S. pneumoniae* available in the NCBI database carry an Rgg0939-homologous protein with >86% identity (Table 1). Remarkably, Rgg2 and Rgg3 in *S. pyogenes* (15) and RovS in *S. agalactiae* (16) are conserved in nearly all of the strains reported. In draft genomes of *S. pneumoniae* at the NCBI (7,415 databases), there are 2,343 hits for homologs of Rgg0939 with at least 70% identity and >70% coverage. Among these, there are 2,308 and 1,765 hits with >85 and 90% identity, respectively. This indicates that the system is present in a significant number of pneumococcal strains. Further, relevance is added as interspecies communication has been shown to occur in Rgg/SHP systems (21, 22). SHP3 in *S. pyogenes* is, in fact, identical to SHP0939 produced by *S. pneumoniae* and only one amino acid different from the SHP produced by *S. agalactiae*. This pheromone has been shown to allow cross-communication with *S. agalactiae* and even with *S. mutans*, which has an SHP with a more divergent sequence (21).

10.1128/mSphere.00324-17.1 FIG S1  Map of *rgg* locus of *S. pneumoniae* D39. (A) On the basis of previous DNA-binding studies (16, 17), the predicted −10 element is in bold and the ribosome-binding site in P*shp* is underlined. The red arrows represent 11 genes downstream of the peptide gene. (B) Alignment of promoter regions of *shp* from *S. pneumoniae*, *shp3* and *shp2* from *S. pyogenes*, and *shp1555* from *S. agalactiae*. Identical bases are gray, and −10 regions are in bold. (C) Similarity of the peptides. The most active part of the pheromone, as described previously (23), is in bold. SHP/SHP3 and SHP1555/SHP2 form two pairs of identical pheromones. Download FIG S1, TIF file, 0.6 MB. Copyright © 2017 Junges et al. 2017 Junges et al. This content is distributed under the terms of the Creative Commons Attribution 4.0 International license.

**TABLE 1:** Genes differentially expressed (*P* < 0.05) >2-fold compared to control samples upon sSHP addition

Gene^a^	Accession no.^b^	Expression		Fold change^c^	*P* value	Product^d^
		Without sSHP^c^	With sSHP^c^			
SPD_0940	ABJ53975.1	1.25	193.41	154.92	6.31E-15	UDP-*N*-acetyl-d-mannosaminuronic acid dehydrogenase putative; MnaB^e^
SPD_0946	ABJ54005.1	4.70	126.37	26.90	1.05E-2	Hypothetical protein
SPD_0942	ABJ53781.1	5.09	111.15	21.83	3.20E-05	Xylose isomerase putative
SPD_0943	ABJ55334.1	7.77	153.57	19.76	2.66E-04	Hypothetical protein
SPD_0948	ABJ55285.1	7.49	143.23	19.12	5.06E-7	Carboxylate-amine ligase
SPD_0944	ABJ53767.1	5.67	106.91	18.86	1.48E-02	Transferase
SPD_0945	ABJ54827.1	8.36	129.15	15.45	8.34E-06	AMP-binding enzyme putative
SPD_0941	ABJ54854.1	6.72	98.42	14.65	1.09E-8	GlcNAc-PI de-*N*-acetylase; UDP-4-galactose-epimerase^e^
SPD_0947	ABJ54194.1	11.04	126.12	11.42	4.87E-03	Membrane protein, putative
SPD_0950	ABJ53827.1	17.18	188.70	10.98	5.05E-13	Transporter major facilitator family protein
SPD_0949	ABJ54337.1	12.78	100.06	7.83	7.31E-07	Bacterial transferase hexapeptide (three repeats), putative

a NCBI protein coding gene.

b GenBank.

c mRNA log_2_ reads. The third and fourth columns show the mRNA expression values (log_2_ reads) in control samples without the pheromone and in samples treated with the synthetic pheromone. Each value is the average of data from two sequencing runs derived from two independent biological experiments. Fold changes in the fifth column are the differences between control and treated samples.

d NCBI protein annotation.

e Protein designation.

### The SHP pheromone acts as an autoinducer via Rgg.

**FIG 1:**
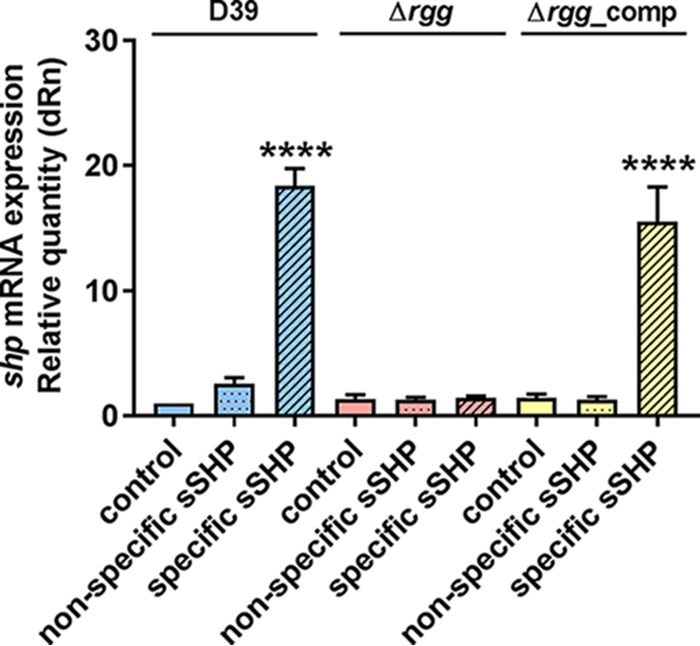
Effect of the sSHP pheromone on relative *shp* expression in *S. pneumoniae* D39 and the Δ*rgg* (SP68), and Δ*rgg*_comp (SP101) mutants. Cultures were grown in C+Y and incubated at 37°C for 2 h at a starting OD_600_ of 0.05 without treatment (control), with the addition of a nonspecific sSHP, or with the addition of the *S. pneumoniae* SHP (specific). The data presented are the means and standard deviation of at least three independent experiments. ****, *P* < 0.0001. dRn, relative mRNA expression.

Pheromone systems most frequently form a positive feedback loop with the regulator, stimulating a fast response and high expression of the pheromone. To investigate whether the putative SHP pheromone sequence upstream of Rgg forms an active autoinducing system, we measured *shp* mRNA expression in cultures treated with the predicted mature pheromone (DIIIIVGG) and in untreated samples. The full ORF in *S. pneumoniae* encodes 23 amino acid residues (MKKISKFLPILFLVMDIIIIVGG), and on the basis of studies of *S. pyogenes* (23), the highest inducing activity is seen in peptides comprising the C-terminal 8 amino acids. Gene expression was nearly 20-fold higher in cultures treated with the specific *S. pneumoniae* pheromone (Fig. 1), indicating that this is indeed a functional autoinducing system.

Rgg/SHP complexes most often activate transcription by binding to the promoter sites that they regulate; however, a previous study showed that the Rgg3 protein of *S. pyogenes* can also function as a transcriptional repressor (15). In this case, once the pheromone is imported into the cell, it binds to Rgg and removes the protein from the repressor position. Given the high similarity of Rgg0939 to Rgg3 of *S. pyogenes*, we investigated whether Rgg0939 would also act as a transcriptional repressor of *shp*. Interestingly, we found that deletion of Rgg0939 does not upregulate *shp* expression (Fig. 1). In addition, treatment of the Δ*rgg0939* mutant strain (SP068) with the synthetic pheromone showed no upregulation of *shp* compared to the control culture, and upon complementation of the strain with *rgg0939* (SP101), upregulation was restored. This indicates that Rgg0939 acts as an activator, rather than as a repressor.

### A single transcript initiated at *shp* and extending to 11 downstream genes is upregulated by the Rgg/SHP signaling system.

**FIG 2:**
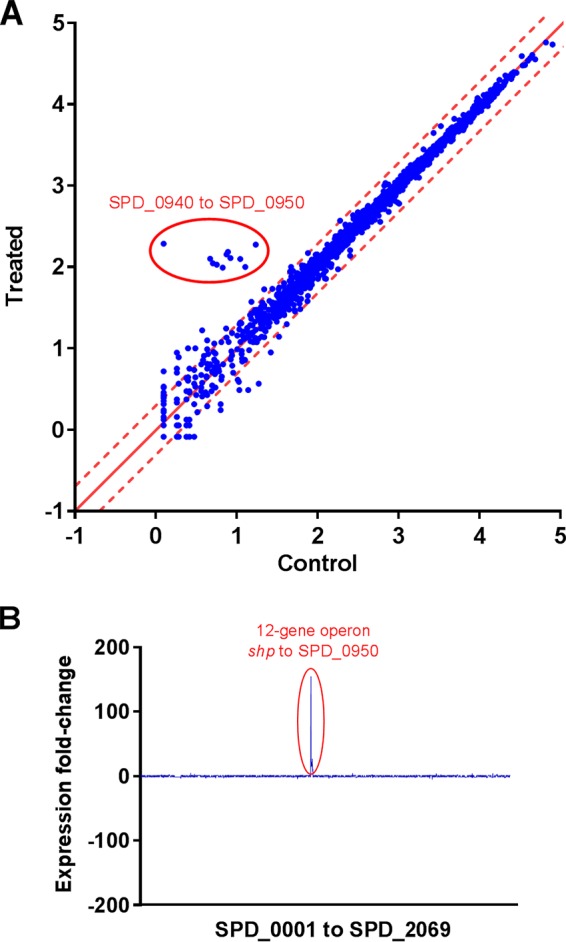
Transcriptome analysis. (A) Scatterplot of global mRNA expression of *S. pneumoniae* D39 with and without addition of the *S. pneumoniae* sSHP (specific). Both axes show mRNA expression (number of reads) on a log_10_ scale. The dashed red lines represent 2-fold up- and downregulation. The operon downstream of *shp* was highly upregulated (circle). Only annotated genes were included in this analysis; therefore, *shp* is not present. (B) Fold change in gene expression in the genome of *S. pneumoniae* D39 when the sSHP is added to the culture. The *x* axis shows gene names in accordance with NCBI Protein Coding Genes. Pseudogenes and tRNA were excluded from the final analyses.

To identify which genes this pheromone system regulates in *S. pneumoniae*, we assessed the transcriptome response in cultures treated with synthetic SHPs (sSHPs) and untreated controls. For selection of optimal conditions for the sSHP response, we tested the expression of *shp* during growth in two different media, tryptic soy broth (TSB) and C+Y (24), by real-time PCR. We found that induction of *shp* by sSHP compared to untreated samples was slightly higher in C+Y than in TSB, particularly at late exponential phase (Fig. S2). The expression of both *shp* and *rgg* increased at the stationary growth phase, which seemed to account for the reduced differences in *shp* expression among treated and untreated samples at this phase. Thus, we decided to collect the samples at late exponential phase by using cultures grown in C+Y. The global mRNA response is shown in Fig. 2.

10.1128/mSphere.00324-17.2 FIG S2  Growth and mRNA expression of *shp* and *rgg* in a D39 culture treated with sSHP over time as relative quantity (dRn) compared to the control. The concentration of sSHP used was 4.5 µM. Bars represent mean values and the standard errors of the means. (A) Growth in TSB and C+Y, The red line represents the control, and the black line represents the SHP-treated culture. (B) mRNA expression of *shp* in TSB and C+Y represented in blue. Pattern-filled bars correspond to SHP-treated samples. (C) *rgg* mRNA expression in TSB and C+Y represented in yellow. Pattern-filled bars correspond to SHP-treated samples. Download FIG S2, TIF file, 0.3 MB. Copyright © 2017 Junges et al. 2017 Junges et al. This content is distributed under the terms of the Creative Commons Attribution 4.0 International license.

**FIG 3:**
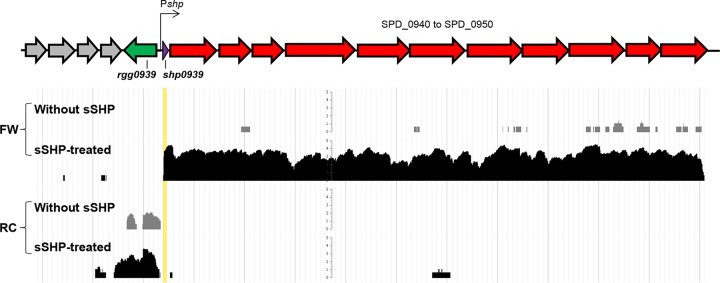
Transcript map of the single 12-gene operon activated by the sSHP. The pheromone is highlighted in yellow. The first row shows the number of reads (log_2_) in the forward (FW) strand (control without sSHP) for nontreated cultures, while the second shows the expression in treated cultures (specific sSHP treated). The third row shows the number of reads (log_2_) in the reverse complement (RC) strand (control without sSHP) for nontreated in comparison to treated *S. pneumoniae* (sSHP treated). Expression values are averages of samples from two independent biological experiments.

A group of 12 genes, comprising 12.7 kb extending from position 949,424 to position 962,125 in the genome of D39, was highly upregulated in the treated culture. The database of prokaryotic operons (DOOR) (25) predicts that the region between SPD_0940 and SPD_0948 forms a single operon, with no candidate terminators, and the MicrobesOnline database predicts an operon from SPD_0940 to SPD_0949 (26). While our data cannot exclude the possibility that they actually form a separate operon, it was clear that upon sSHP stimulation, the transcript extended all the way from *shp* to SPD_0950 (Table 2). The map of mRNA expression levels in this region shows a continuous transcript corresponding to the forward strand, whereas in the control culture without sSHP, this transcript was virtually absent (Fig. 3). The 11 genes downstream of *shp* comprise a variety of functions and annotations, such as MnaB (SPD_0940), UDP-4-galactose-epimerase (SPD_0941), a putative xylose isomerase (SPD_0942), an AMP-binding enzyme (SPD_0945), membrane proteins, and transferases. Homologous genes such as those for MnaB, UDP-4-galactose-epimerase, AMP-binding enzymes, membrane proteins, and transferases were also identified downstream of *shp* in other strains of *S. pneumoniae* (Table 2). In addition, other genes with distinct functions such as lantibiotic and bacitracin transport, lanthionine biosynthesis protein, and lactococcin processing and transport were identified (Table 1).

**TABLE 2:** Rgg homology and presence of putative pheromone SHP in other *S. pneumoniae* strains

Strain	NCBI accession no.	Identity to Rgg0939 (%)^a^	*rgg* accession no.^b^	*shp*?	Sequence	Downstream of peptide^c^
R6 (D39 derivative)	NC_003098.1	Yes (100)	spr0960	Yes	MKKISKFLPILFLVMDIIIIVGG	Potential 12-gene operon identical to D39; 1st gene (spr0961) encodes UDP-*N*-acetyl-d-mannosaminuronic acid dehydrogenase, 9th (spr0969) encodes nikkomycin biosynthesis protein
JJA	NC_012466.1	Yes (97)	SPJ_0996	Yes	MKKISKFLPILVLVMDIIII	Potential 3-gene operon followed by 2nd 3-gene operon; 2nd gene of 1st sequence (SPJ_0998) is annotated as lantibiotic mersacidin transporter system, 1st gene of 2nd operon (SPG_1000) is annotated as bacitracin transport ATP-binding protein (BcrA)
A45^d^	NC_018594.1	Yes (96)	SPNA45_01354	Yes	MKKISKFLPILVLVMDIIII	Potential 3-gene operon followed by 2-gene operon; 1st gene (SPNA45_01355) encodes lanthionine biosynthesis protein (LanM), 2nd (SPNA45_01356) encodes toxin secretion ABC transporter, 1st gene of 2nd operon (SPNA45_01358) encodes BcrA
		Yes (86)	SPNA45_00934	Yes	MEKISKFLPILVLVMDIIIIVGG	Potential 4-gene operon; 1st (SPNA45_00935) encodes pyridoxal-dependent decarboxylase, 3rd (SPNA45_00937) encodes long-chain fatty acid CoA ligase, 4th (SPNA45_00938) encodes macrolide efflux ABC transporter permease
A66^d^	NZ_LN847353.1	Yes (96)	A66_00963	Yes	MKKISKFLPILVLVMDIIII	Potential 3-gene operon followed by 2-gene operon very similar in organization to SPNA45_01354
		Yes (87)	A66_01135	Yes	MKKISKFLPILVLVMDIIIIVGG	Potential 4-gene operon; 1st gene downstream (A66_01134) encodes siderophore biosynthesis decarboxylase, 2nd encodes nonribosomal peptide synthetase, 4th encodes membrane-spanning ABC transporter
NT_110_58	NZ_CP007593.1	Yes (95)	SpnNT_00981	Yes	MKKISKFLPILVLVMDIIII	Potential 7-gene operon; 1st gene (SpnNT_00982) encodes LanM, 2nd encodes lactococcin G-processing and transport ATP-binding protein (LagD), 3rd (SpnNT_00984) encodes alpha-hemolysin translocation ATP-binding protein (HlyB)
ATCC 700669	NC_011900.1	Yes (94)	SPN23F09790	Yes	MKKISKFLPILVLVMDIIII	Potential 2-gene operon followed by 3-gene operon; 1st gene (SPN23F_09810) encodes lantibiotic export protein, 3rd (SPN23F_09830) encodes lantibiotic transport ATP-binding protein
P1031	NC_012467.1	Yes (91)	SPP_1060	Yes	MKKISKFFPILMLVMDIIIIVGG	Potential 7-gene operon; 1st gene (SPP_1062) encodes NAD-binding domain of 6-phosphogluconate dehydrogenase family, 6th (SPP_1067) encodes UDP-glucose 4-epimerase (GalE1), 7th (SPP_1068) encodes biotin carboxylase
NCTC7465	NZ_LN831051.1	Yes (91)	ERS445053_01678	Yes	MKKISKFFPILMLVMDIIIIVGG	Potential 7-gene operon very similar to SPP_1060
gamPNI0373	NC_018630.1	Yes (91)	HMPREF1038_01125	Yes	MKKISKFFPILMLVMDIIIIVGG	Potential 7-gene operon very similar to SPP_1060
AP200	NC_014494.1	Yes (89)	SPAP_1144	Yes	MKKISKFFPILMLVMDIIIIVGG	Potential 2-gene operon with unknown function; immediately downstream there are 3 other operons composed of 4, 2, and 3 genes, respectively
G54^d^	NC_011072.1	Yes (88)	SPG_0976	Yes	MKKISKFFPILMLVMDIIIIVGG	Potential 2-gene operon followed by 7-gene operon; last 2 genes (SPG_0984, SPG_0985) are ABC transporter permeases; SPG_0982 encodes UDP-glucose 4-epimerase
		Yes (34)	SPG_1268	Yes	MKKYYQIFLLLFDIIIIGLYQ	Potential 4-gene operon with unknown function
670–6 B	NC_014498.1	Yes (36)	SP670_1149	Yes	MKKYYQIFLLLFDIIIIGLYQ	Potential 4 operons composed of 2, 1, 2, and 2 genes, respectively; all encode hypothetical proteins except 2nd gene (SP670_1147), which encodes PcfD (bacteriocin ABC-type transporter)
INV200	NC_017593.1	No				
CGSP14	NC_010582.1	No				
TIGR4	NC_003028.3	No				
70585	NC_012468.1	No				
Taiwan19F-14	NC_012469.1	No				
Hungary19A-6	NC_010380.1	No				
TCH8431/19A	NC_014251.1	No				
OXC141	NC_017592.1	No				
INV104	NC_017591.1	No				
SPN034156	NC_021006.1	No				
SPN034183	NC_021028.1	No				
SPN994038	NC_021026.1	No				
SPN994039	NC_021005.1	No				
SPN032672	NC_021003.1	No				
SPN033038	NC_021004.1	No				
ST556	NC_017769.2	No				
SP49	NZ_CP018136.1	No				
SP61	NZ_CP018137.1	No				
SP64	NZ_CP018138.1	No				
SWU02	NZ_CP018347.1	No				

a Hits with >30% identity with Rgg0939 in an NCBI tBLASTn search were considered.

b NCBI accession numbers for protein coding genes.

c Operon prediction was conducted with DOOR (35).

d Strains G54, A66, and SPNA45 have two proteins similar to Rgg0939.

**FIG 4:**
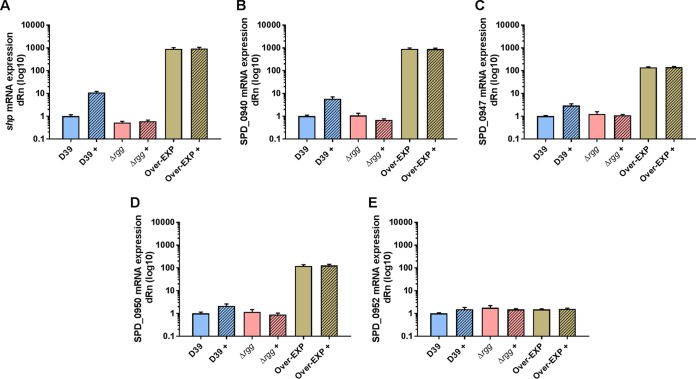
Activation of *shp* and the downstream regulon with and without sSHP in *S. pneumoniae* D39 and the Δ*rgg* (SP68) and Over-EXP (SP44) mutant strains. *shp* gene expression was determined by RT-PCR. Cultures were grown in C+Y and incubated at 37°C for 2 h at a starting OD_600_ of 0.05 with (as indicated by a plus sign) or without 1 µM *S. pneumoniae* sSHP prior to RNA extraction. The *y* axis shows relative mRNA expression (dRn) on a log_10_ scale. Each bar represents mean values with the standard error of the mean. Panels: A, *shp*; B, SPD_0940; C, SPD_0947; D, SPD_0950; E, SPD_0952.

To further investigate the function of the Rgg/SHP regulon, we created an overexpression mutant (SP044) by deleting the regulator *rgg0939* and inserting a kanamycin resistance cassette without a terminator into the opposite strand (Fig. S3). We confirmed the expected expression profile of the Δ*rgg* (SP068) and Over-EXP (SP044) mutants by reverse transcription (RT)-PCR with specific primers for *shp*, SPD_0940, SPD_0947, and SPD_0950 (Fig. 4). No growth differences between the mutant and wild-type strains were observed in three different media, TSB, C+Y, and CDM++ (27) (Fig. S4).

10.1128/mSphere.00324-17.3 FIG S3  Diagram of the different characteristics of the two mutants (SP68 and SP44) utilized in this study. Download FIG S3, TIF file, 0.1 MB. Copyright © 2017 Junges et al. 2017 Junges et al. This content is distributed under the terms of the Creative Commons Attribution 4.0 International license.

10.1128/mSphere.00324-17.4 FIG S4  Growth of wild-type D39 and the Δ*rgg* (SP68) and Over-EXP (SP44) mutants in three different media. Precultures were diluted 1:10 in fresh medium and divided into 200-µl wells for OD_600_ measurements every 15 min for 7 h. Panels: A, TSB; B, C+Y; C, CDM++. Download FIG S4, TIF file, 0.1 MB. Copyright © 2017 Junges et al. 2017 Junges et al. This content is distributed under the terms of the Creative Commons Attribution 4.0 International license.

### Production of surface polysaccharides is increased in the overexpression mutant.

**FIG 5:**
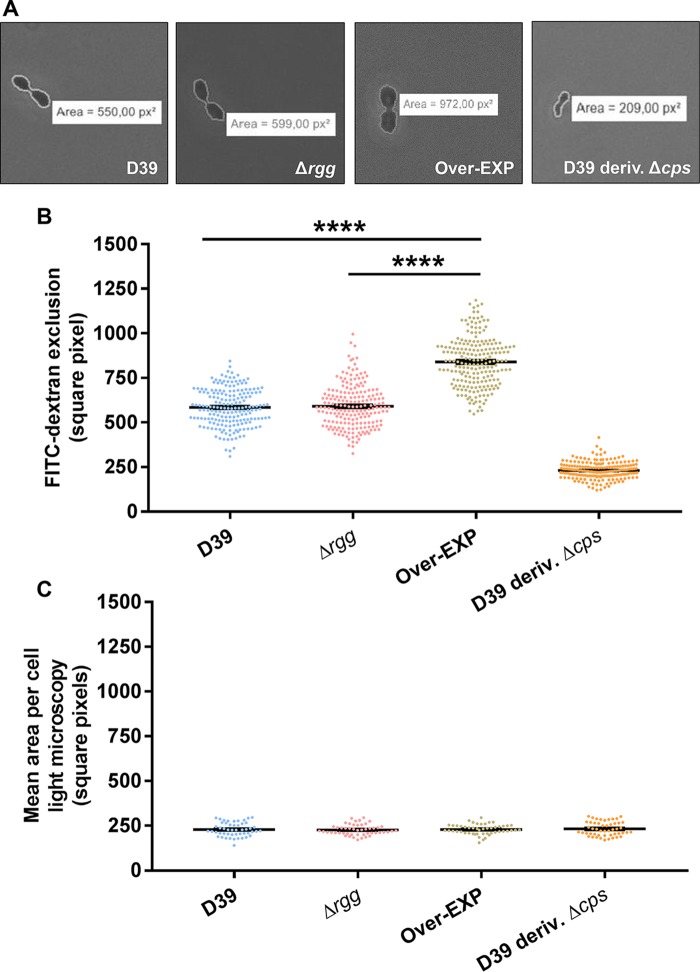
Overexpression of the Rgg0939 regulon affects serotype 2 capsule thickness. Each dot represents the measurement of the FITC-dextran exclusion area for a single cell, and each bar represents the mean and the standard error of the mean of each group. (A) Representative microscopic images of FITC-dextran exclusion for D39 and the Δ*rgg* and Over-EXP mutant strains. (B) FITC-dextran analysis of capsule thickness. The mean area (square pixels) per bacterium was greater in the overexpression strain than in D39 and the Δ*rgg0939* mutant. ****, *P* < 0.0001. (C) Light microscopy analysis of cell size (square pixels).

The upregulation of genes putatively involved in polysaccharide biosynthesis upon the addition of sSHP led to the hypothesis of a possible role for the Rgg/SHP pheromone system in surface polysaccharide production by *S. pneumoniae* D39. Among the 11 genes in the Rgg0939 regulon, 2 (SPD_0940 and SPD_0941) seem to have a putative role in polysaccharide production on the basis of a homology search. SPD_0940 was ∼155-fold upregulated by sSHP and presents 27.2% identity to the gene encoding the enzyme MnaB, an essential component of the MnaA/MnaB biosynthetic pathway in pneumococcal capsule production of serotypes 12F and 12B. Furthermore, it has 26% identity to a gene in the capsule (*cps*) operon of *Streptococcus suis* (*cps3N*). SPD_0941 presented an upregulation of ∼15-fold, and a homology search revealed 30% identity to GalE of *Escherichia coli*, a UDP-galactose-4-epimerase that interconverts UDP-glucose (UDP-Glc) and UDP-galactose (UDP-Gal) and is present in the *cps* locus of *Staphylococcus aureus* serotypes 5 and 8 (28). The serotype 2 *cps* locus encodes five type-specific transferases, one initiating transferase that adds the first sugar to a lipid receptor (*cps2E*) and four glycosyltransferases (*cps2T*, *cps2F*, *cps2G*, and *cps2I*) that further link sugars to create repeat units. The two putative transferases present in the Rgg0939 regulon (SPD_0944 and SPD_0949) seemingly do not present homology with the glycosyltransferases present in the *cps* locus; however, they are similar to enzymes present in *cps* loci of *E. coli* (*wbbj*, 36% identity) and *S. agalactiae* (*neuD*, 31% identity). To examine whether the Rgg/SHP system is involved in surface polysaccharide production, we used the fluorescein isothiocyanate (FITC)-dextran assay, where the zone of exclusion of FITC-dextran indicates polysaccharide thickness (29–34). A D39 derivative strain without the capsule (R36A) was included as a negative control. R36A originates from experiments reported by Avery et al. (35) and was obtained by 36 serial passages of D39 growing in the presence of anti-type 2 rabbit serum. The sequence of the capsule locus in this strain shows a deletion of ∼7.5 kb, including the first nine genes of the capsule operon (*cpsA*BCDETFGH) (36). The area occupied by R36A, as determined by light microscopy, was similar to the calculated area in the FITC-dextran assay using fluorescence microscopy. This confirmed that under the conditions used, no surface polysaccharide was produced by the unencapsulated strain (Fig. 5). The exclusion area was larger for D39, as expected for an encapsulated strain, and was not different from that of the Δ*rgg0939* mutant. In contrast, a significantly larger mean area of FITC exclusion per cell was observed with the overexpression strain (Fig. 5). The results indicate that the overexpression strain produces a thicker surface-associated polysaccharide, providing support for the hypothesis that the Rgg/SHP-regulated genes are involved in surface polysaccharide production.

### Inactivation of the Rgg/SHP system enhances biofilm formation, whereas overexpression of the Rgg regulon has the opposite effect.

**FIG 6:**
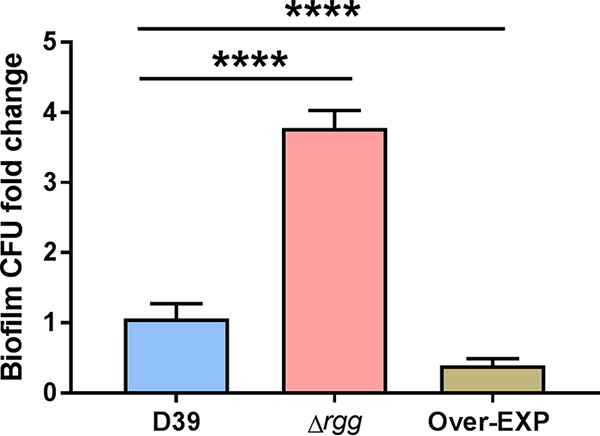
Biofilm formation of wild-type D39 and Δ*rgg* (SP68) and Over-EXP (SP44) mutants on eukaryotic cells. Cultures were grown in CDM++ to an OD_600_ of 0.1 and inoculated on top of a fixed lung cell line (A549). Every 12 h, the medium was changed to avoid bacterial lysis. At 36 h, the supernatant was removed, the biofilm was resuspended in rich medium, the wells were scraped, and the material obtained was immediately refrigerated for plating. The *y* axis shows the number of biofilm CFU recovered from wells as fold change compared to wild-type D39, and each value is the mean and the standard error of the mean of at least three independent biological experiments. ****, *P* < 0.0001.

In *S. pneumoniae*, the capsule is thought to block surface proteins important for adhesion, thus impairing biofilm formation (37). To investigate whether the Rgg regulon, which is involved in the synthesis of surface polysaccharides, as demonstrated above, is involved in biofilm formation, we tested the ability of D39 and the deletion and overexpression mutants to form biofilms by using a eukaryotic lung cell line (A549) as the substrate. The overexpression strain formed less biofilm on the biotic surface than D39 did (Fig. 6), while the Δ*rgg* mutant formed significantly more. Such findings corroborated our hypothesis that overexpression of the regulon would impair biofilm formation because of the increased production of surface polysaccharides. Also, the difference in biofilm formation between the *rgg* deletion mutant and D39 indicates that the system is active under biofilm growth conditions.

### Overexpression of the Rgg/SHP regulon impairs fitness in a mouse model of lung infection.

**FIG 7:**
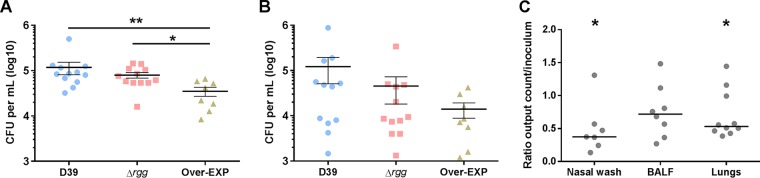
Bacterial burden of wild-type D39 and Δ*rgg* (SP68) and Over-EXP (SP44) mutant strains in a mouse early lung infection model. Each dot represents results for a single mouse, and bars represent the mean and the standard error of the mean of each group. Mice were inoculated intranasally with 5 × 10^6^ CFU in 50 µl of 1× PBS and killed after 24 h. Recovery of samples took place immediately after death, and all washes were placed at 4°C for dilution and plating. (A) Nasal cavity wash. **, *P* < 0.01; *, *P* < 0.05. (B) BALF. (C) Mixed-infection assay with D39 and Over-EXP. The first set was recovered from nasal wash. Each point represents results for a single mouse, and bars show the median CI of the group. The second and third sets show bacteria recovered from BALF and mashed lungs, respectively. The *y* axis shows the ratio of the bacterial counts recovered to the inoculum. A ratio of >1 indicates that the overexpression strain was predominant, while a ratio of 0 to 1 indicates that D39 was recovered in higher numbers. *, *P* < 0.05.

We further asked if changes in surface polysaccharide expression could also affect *S. pneumoniae* fitness in the host. We observed that the overexpression strain had a reduced ability to cause early lung disease (Fig. 7). To assess if the impaired ability of the overexpression strain to form biofilms and cause disease could be affected by its fitness compared to that of the parent strain (38), we inoculated mice with D39 and the overexpression strain. The overexpression strain was recovered in lower numbers from nasal washes than D39 (Fig. 7C). Overall, competitive assays indicate that overexpression of the Rgg/SHP regulon reduces *S. pneumoniae* fitness in a murine model of lung infection. In contrast to the overexpression mutant, the deletion strain was not significantly affected in virulence.

### SHP stimulates exopolysaccharide production in unencapsulated strain R36A.

**FIG 8:**
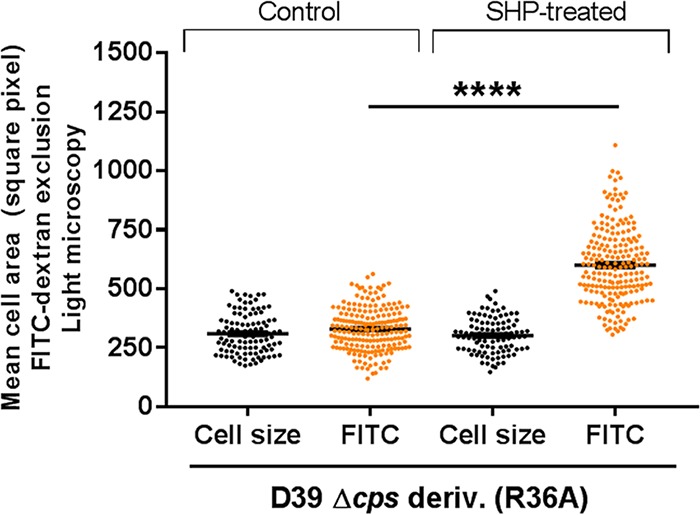
Production of surface polysaccharides following treatment with synthetic SHP. Each dot represents the measurement of the FITC-dextran exclusion area for a single cell, and bars represent the mean and the standard error of the mean of each group. ****, *P* < 0.0001.

We next asked if treatment with sSHP could increase the production of surface polysaccharides and whether such an effect would depend on the presence of a functional capsule locus. We treated D39, an unencapsulated D39 derivative strain (R36A), and the Δ*rgg* and Over-EXP mutant strains with a specific and a nonspecific sSHP and measured both cell size and FITC-dextran exclusion in all samples. No differences in surface polysaccharide expression were observed in any of the strains when a negative-control nonspecific sSHP peptide from *Streptococcus salivarius* was added to the cultures. Differences in polysaccharide thickness were also not detected after the addition of a specific sSHP to D39 or the derivative Δ*rgg* and Over-EXP mutants (Fig. S5). In untreated R36A, the cell size matched the FITC-dextran exclusion area, as expected; however, upon treatment with the specific sSHP, the FITC-dextran exclusion area increased significantly, reaching the levels of encapsulated D39 (Fig. 8). Such findings corroborate the above-described data showing that the new locus participates in the regulation of surface polysaccharides in *S. pneumoniae* and that this increase is observed even in the absence of a functional serotype 2 capsule locus.

10.1128/mSphere.00324-17.5 FIG S5  Effects of a negative-control peptide and SHP on capsule thickness. Each dot represents a single-cell measurement of FITC-dextran exclusion. Bars represent the mean and the standard error of the mean of each group. The mean area (square pixels) of the R36A strain was increased by the addition of sSHP. Download FIG S5, TIF file, 0.2 MB. Copyright © 2017 Junges et al. 2017 Junges et al. This content is distributed under the terms of the Creative Commons Attribution 4.0 International license.

## DISCUSSION

**FIG 9:**
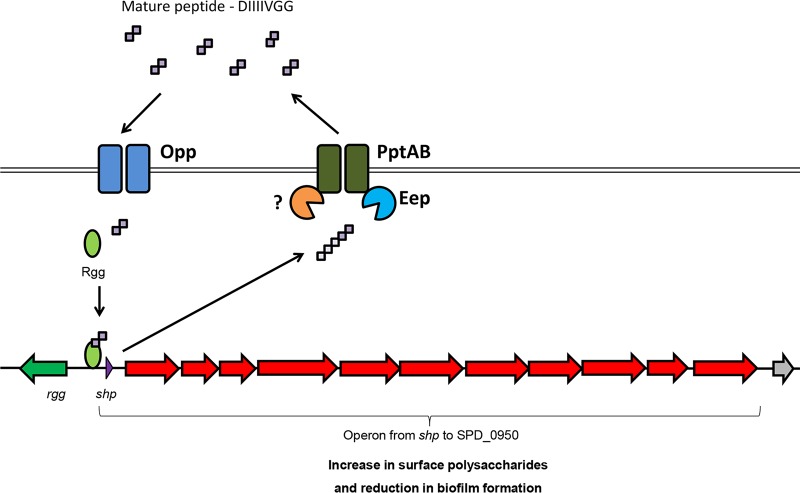
Schematic representation of the Rgg/SHP system in *S. pneumoniae* D39. The SHP precursor is produced, processed, and exported by PptAB and Eep (16, 77). Once it reaches the quorum-sensing threshold, the mature peptide is imported by Opp (12, 15, 16), and it binds to Rgg for activation of the *shp* promoter. This, in turn, activates the pheromone positive-feedback loop and an operon formed by 12 genes initiated at the *shp* gene. High activation of this regulon upregulates polysaccharide synthesis and downregulates biofilm formation.

The pneumococcus has been a model organism for the major discoveries regarding natural transformation, culminating with the discovery of DNA as hereditary material (35, 39). The discoveries gradually progressed to the description of a competence factor in culture supernatants as the first report of quorum sensing in Gram-positive bacteria (40, 41). More recently, Rgg proteins, once seen as exclusive stand-alone regulators, were identified together with SHPs as components of newly described quorum-sensing signaling systems found in the majority of streptococci (12, 13). Studies of the Rgg/SHP systems in *S. pyogenes* and *S. agalactiae* have revealed that while the mechanisms involved in SHP production and processing are similar, the regulated genes differ (15, 16), indicating that the system has evolved to control different social behaviors. Here we present for the first time the characterization of an Rgg/SHP system in *S. pneumoniae*. By using directional transcriptome sequencing (RNA-Seq), which provides information on the direction of transcripts, this study provides the most detailed map of a transcriptome capturing Rgg/SHP responses, and it shows that one single transcript initiated at *shp* is regulated by this system. In this transcript, different genes carrying annotations related to polysaccharide synthesis were identified. By stimulating the expression of the *rgg* regulon, an increase in surface polysaccharide expression was produced, concomitant with a decrease in biofilm formation (Fig. 9).

An emerging theme in social behaviors controlled by quorum sensing is the regulation of surface polysaccharide expression. The first Rgg regulator identified in streptococci, although not associated with any known quorum-sensing peptide signal, was indeed found to control the expression of a glucosyltransferase in *Streptococcus gordonii* (42). In *Pseudomonas aeruginosa*, acyl homoserine lactone signals activate, for instance, a cascade of events that, although indirect, lead to increased expression of matrix polysaccharides (43). Direct regulation of polysaccharide synthesis genes has also been described, such as in the Gram-negative bacterium *Rhodobacter capsulatus*, in which capsule production is controlled by intracellular regulators that bind homoserine lactone quorum-sensing signals (44). The connection between quorum sensing and extracellular polysaccharide production has also been observed for regulators in the bacteria *Pantoea stewartii* and *Vibrio vulnificus* that act as repressors of capsule synthesis (33, 45). Interestingly, in the Rgg system of *S. pyogenes*, genes with homology to polysaccharide synthesis genes seem to be activated by SHP (15), but their functions remain mostly uncharacterized. In this study, overexpression of the Rgg target regulon in D39 and stimulation with sSHP in the derivative unencapsulated strain R36A led to increased exopolysaccharide expression. Interestingly, in the D39 wild type, no stimulation by sSHP was observed, which contrasted with the results obtained with the overexpression mutant. This is most probably due to the levels of stimulation of the putative polysaccharide expression genes of the Rgg regulon, which were considerably lower in D39 than in the overexpression mutant.

*S. pneumoniae* exhibits highly variable capsules, with >90 pneumococcal serotypes currently recognized that vary in polysaccharide composition and structure from linear repeats of two sugars (serotype 3) (46) to highly complex repeats of multiple sugars with side branches (serotype 12F) (47). The strain used in this study, *S. pneumoniae* D39 serotype 2, contains six sugars per repeat unit (three rhamnose residues, one glucuronic acid, and two glucose units), and the polymerizing linkage happens between the first and fourth sugars (47, 48). The biosynthetic pathway encoded specifically by the *cps* loci of serotype 2 takes part via UDP-glucose-6-dehydrogenase, a key compound in the biosynthesis of pneumococcal capsules that converts the precursor sugar substrate UDP-glucose (UDP-Glc) into UDP-glucuronic acid (UDP-GlcA) (36, 49, 50). In contrast, serotypes 12F and 12A employ another biosynthetic pathway for capsule production by using the enzymes MnaA and MnaB. Our data show that the Rgg0939 regulon encloses a homolog of MnaB, which is responsible for the conversion of *N*-acetylmannosamine (product of a previous reaction catalyzed by MnaA) into *N*-acetylmannosaminuronic acid (UDP-ManNAcA), an uncommon capsule component present only in serotypes 12F and 12A (48).

It is intriguing, however, that (i) UDP-ManNAcA has not been identified in the capsule structure of D39, (ii) D39 only produces MnaB, and (iii) the gene responsible for MnaB production is located outside the capsule loci. On the other hand, it has been previously noted that essential enzymes involved in capsule production are not seldom encoded in a locus other than the one involved in capsule regulation. One example is GalU, one of the proteins required for adequate production of UDP-Glc, the main serotype 2 capsule precursor (51, 52). Furthermore, GalE, a UDP-4-glucose-epimerase, has been shown to be involved in lipopolysaccharide synthesis in Gram-negative bacteria. UDP-Glc is the initial substrate of the serotype 2 capsule; thus, it is likely that GalE converts UDP-Gal into UDP-Glc, increasing its availability for subsequent polysaccharide production. Although this conversion has not been shown experimentally in *S. pneumoniae* (53), cells can be metabolizing exogenous galactose as a primary energy substrate for capsule synthesis. In addition, galactose has been recently suggested to be a possible component of the exopolysaccharide matrix formed by *S. pneumoniae* unencapsulated strain R6, which is a derivative of R36A (54). This can partially explain the increased capsule thickness of the overexpression strain and SHP-stimulated unencapsulated strain R36A. Further studies are under way to investigate the nature of the surface polysaccharide regulated by the identified *S. pneumoniae* SHP/Rgg system.

Extracellular polysaccharides, represented by capsule structures, as well as by loosely associated exopolysaccharides and other forms of polysaccharide structures, have long been known to affect biofilm formation. In *S. pneumoniae*, capsule expression has been negatively associated with biofilm formation (55), whereas other forms of polysaccharides seem to promote biofilm formation (54). The inhibition of biofilm formation by the capsule is attributed to the capsule effect on blocking the exposure of *S. pneumoniae* surface adhesins that promote attachment to epithelial cells (37). Our results showed that the biofilm phenotype of the *rgg* deletion mutant deficient in quorum sensing resembles that of capsule deletion mutants of *S. pneumoniae* and the close relative *Streptococcus mitis*, which form thicker biofilms on epithelial cells in the absence of the capsule (55). Comparing the results obtained with those of the dextran exclusion assay, which uses planktonic cells and showed no differences between D39 and the *rgg* deletion mutant, with the biofilm results showing remarkable differences in biofilm, the results suggest that planktonic conditions do not favor expression of the Rgg regulon. This is further reinforced by the fact that placing the Rgg regulon under the control of a constitutive promoter supporting overexpression of the regulon resulted in reduced biofilm formation. Of note, in this study, biofilm formation was assessed by comparing the mean total numbers of CFU recovered from experiments.

Previous reports have shown a positive correlation of capsule expression and virulence in *S. pneumoniae* (29, 56). In animal models, unencapsulated strains, in general, show a reduced capacity to colonize and cause infections. Most important, though, seems to be the ability of *S. pneumoniae* to regulate capsule expression. In line with this, Hammerschmidt et al. (37) showed that pneumococci attached to or invading lung epithelial cells downregulate their capsule, minimizing the physical interference for successful adhesion. Also, a recent study showed that both over- and underexpression of the capsule locus reduced *S. pneumoniae* fitness in infection models (57). While no difference was observed between D39 and the *rgg* deletion mutant, which may reflect functional redundancy because of the expression of the original capsule, the overexpression strain was recovered in lower numbers from the nasal cavity and bronchoalveolar lavage fluid (BALF). Such an outcome might be explained by a high metabolic cost (number of carbons and high-energy bonds) involved in the production of capsular polysaccharide (29) and the inability to fine-tune capsule expression (57). Remarkably, surface polysaccharides serve several functions for bacteria, including but not limited to escape from phagocytosis by macrophages and neutrophils and protection or increased sensitivity to human defensins (58–60). Thus, the identified Rgg/SHP quorum-sensing-regulated behavior is likely to have implications beyond those characterized here.

Quorum-sensing signaling of Rgg/SHP systems is often found to be inhibited or decreased in peptide-rich media (16, 61). In this study, stimulation of the quorum-sensing system was similar in rich, semidefined and defined media. It is important to note, however, that the saturation threshold point in each particular medium was not assessed and that further optimization of experimental conditions may demonstrate different levels of stimulation.

The mature form of the *S. pneumoniae* D39 SHP (DIIIIVGG) is identical to SHP3 of the Rgg2/3 system in *S. pyogenes* and to SHP1044 in *S. thermophilus*. Together with the finding that even slightly divergent sequences can promote interspecies cross-communication (21, 22), it is possible that the Rgg/SHP system has an important role in microbial interactions taking place in multispecies communities. Finally, the finding that *S. pneumoniae* D39 uses a quorum-sensing system to directly regulate a single locus with a role in surface polysaccharide expression suggests an important function for the system in adaptation and survival. The importance of controlling surface polysaccharide production as a collaborative group behavior is supported by genome analysis showing homologs of this system in other *S. pneumoniae* strains that have the Rgg/SHP system and by the link between quorum sensing and capsule production in other bacterial species distantly related to streptococci.

## MATERIALS AND METHODS

### Bacterial strains and media.

The *S. pneumoniae* strains used in this study are listed in Table 3. All strains were stored at −80°C in Todd-Hewitt broth (THB; Becton, Dickinson and Company, Le Pont de Claix, France) supplemented with 0.5% yeast extract and 30% glycerol. Precultures were prepared as described previously (62). For growth, RNA sequencing, and biofilm experiments, the media used were TSB (Bacto soybean-casein digest medium), THB, C+Y (24), and a chemically defined medium supplemented with 1% TSB and 0.5% choline (CDM++) (27). For plates, blood agar base number 2 (Oxoid, Hampshire, England) supplemented with 5% defibrinated sheep blood (TCS Biosciences Ltd., Buckingham, United Kingdom) was used. The selective antibiotic used was kanamycin at 500 μg·ml^−1^.

**TABLE 3:** Strains used in this study

Strain	Description	Source
NCTC7466	*S. pneumoniae* D39	NCTC^a^
NCTC10319	*S. pneumoniae* R36A	NCTC
SP068	NCTC7466 but Δ*rgg0939*	This study
SP044	NCTC7466 but Δ*rgg0939*::Kan; Kan^r^ Over-EXP	This study
SP101	SP068 complemented with *rgg0939*	This study

a NCTC, National Collection of Type Cultures (Central Public Health Laboratory, London, England).

### Pheromones.

The predicted specific *S. pneumoniae* mature pheromone SHP (NH_2_-DIIIIVGG) was obtained from the GenScript Corporation (NJ) as a custom synthetic peptide and stored as a sterile 10 mM solution in 3% ammonia water at −20°C. Stock solutions of lyophilized CSP-1 were prepared by resuspending it in distilled water to a concentration of 10 mM and storing it at −20°C. Working solutions of 1 and 0.1 mM were aliquoted and stored at −20°C. As a negative-control peptide, SHP from *S. salivarius* (NH_2_-PYFTGCL) was synthesized (GenScript Corporation, NJ) and stored as a 10 mM solution at −20°C.

### Bacterial growth.

For bacterial growth assays, precultures of the strains were centrifuged at 8,000 × *g* at 4°C for 8 min; suspended 1:10 in TSB, C+Y, or CDM++; and transferred in 200-µl volumes to wells of a 96-well plate. Optical density at 600 nm (OD_600_) was measured every 15 min at 37°C.

### Real-time PCR.

Precultures were centrifuged at 8,000 × *g* at 4°C for 8 min and resuspended 1:10 in TSB, C+Y, or CDM++, and cultures were incubated at 37°C in 5% CO_2_ for 20 min. Cultures were divided in two, with one half being treated with sSHP and the other half being treated with the same volume of 1× phosphate-buffered saline (PBS). Cultures were incubated for different times at 37°C and 5% CO_2_ and harvested by centrifugation at 8,000 × *g* at 4°C for 8 min. RNA was extracted with the High Pure RNA Isolation kit (Roche). The First Strand cDNA Synthesis kit from Thermo Scientific (Fermentas) was used in accordance with the manufacturer’s instructions. The primers used are listed in Table S1.

10.1128/mSphere.00324-17.7 TABLE S1  Primers used for RT-PCR and construction of mutants. Download TABLE S1, DOCX file, 0.02 MB. Copyright © 2017 Junges et al. 2017 Junges et al. This content is distributed under the terms of the Creative Commons Attribution 4.0 International license.

### RNA-Seq sample preparation and analysis.

*S. pneumoniae* precultures were centrifuged at 8,000 × *g* at 4°C for 8 min and resuspended 1:10 in C+Y; 10-ml culture volumes were incubated at 37°C at 5% CO_2_ for 20 min; and the cultures were divided in two, with one half being treated with sSHP (1 µM, N-DIIIIVGG) and the other half being treated with the same volume of 1× PBS. Cultures were incubated for 2 h at 37°C in 5% CO_2_ and harvested at 8,000 × *g* at 4°C for 8 min. RNA was extracted with the *mir*Vana microRNA isolation kit (Life Technologies, Inc.). After a first extraction, lysed cells were treated with TURBO DNase (Life Technologies, Inc.) and a second RNA extraction was performed. RNA quality was tested on a bioanalyzer with the RNA 6000 Nano kit. The 16S and 23S rRNAs were then removed with the MICROBExpress Bacterial mRNA Enrichment kit (Thermo Fisher Scientific). RNA was treated with the NEXTFlex Directional RNA-Seq kit (dUTP based) for preparation of the DNA library for paired-end sequencing with Illumina HiSeq by the Norwegian Sequencing Centre (http://www.sequencing.uio.no). A sequence in FASTQ format was derived from each sample. Analyses were performed in accordance with previously reported protocols (63, 64).

### Construction of mutants with antibiotic resistance markers.

Knockout deletion mutants were constructed by the PCR mutagenesis method described previously (65). All of the strains constructed and the primers used are listed in Table S1.

### High-efficiency transformation and markerless mutant construction.

Encapsulated pneumococcal strains present very low *in vitro* transformation efficiency levels (66–69). While low efficacy can still be used for mutant construction carrying markers, for direct genome editing, higher transformation efficiencies are valuable. Thus, to create markerless mutants of D39, we adapted the growth conditions, synthetic peptide, medium, and screening protocol from a recently developed strategy that we optimized for *S. mitis*, a close relative of *S. pneumoniae* (62), which resulted in increased efficiency rates of 4 to 21%. This improvement in transformation efficiency allowed for direct markerless deletion of *rgg0939* from *S. pneumoniae* D39. Table S2 contains details of how we assembled the previously described C+Y_YB_ medium (70). An amplicon with 3-kb flanking regions was constructed by overlapping PCR and transformed into D39 in accordance with the protocol for high transformation efficiency shown in Table S3. Initial screening of 40 colonies (Fig. S6A) showed two positive colonies; one was a mixture of both mutant and parent alleles, and the other was a pure mutant. The pure colony was then selected, grown for another 6 h at 37°C, and replated. On the next day, three derivate colonies of this isolate were tested with two sets of primers, positive (FP952/FP1126) and negative (FP952/FP1579) selection, for confirmation of the mutation (Fig. S6B). After confirmation, cultures were stocked at −80°C supplemented with 30% glycerol. Strain SP068 was complemented with *rgg0939* (SP101) by using a markerless amplicon carrying the gene (FP1577/FP1578 amplified from D39). Selection and confirmation of the mutation were performed by screening PCR with two sets of primers, FP952/FP1126 and FP952/FP1579, for confirmation of the mutation.

10.1128/mSphere.00324-17.6 FIG S6  Colony genotyping of *S. pneumoniae* D39 and the Δ*rgg* (SP68) and Over-EXP (SP44) mutant strains. (A) Forty colonies were screened with primers FP952 and FP1126. One colony presented only the mutant allele (SP68) (lane M, 530-bp band), while other presented both parent and mutant alleles (lane X, 1,304- and 530-bp bands). Thirty-eight colonies presented only the parent allele (lane P, 1,304-bp band). The transformation efficiency was 5%. Lanes L, DNA ladder Gene Ruler 1 kb (GenScript). *, positive control with D39 DNA. **, negative control with TIGR4 DNA. Lane M, mutant colony (SP68). Lane P, parent strain colony (D39). Lane X, mixed colony presenting both parent and mutant alleles. (B) Three colonies (C1 to C3) were isolated from the mutant represented in lane M and grown for several generations to promote segregation of the parent (D39) and mutant (Δ*rgg*) alleles. Primer pair 1 (FP952/FP1126) amplifies a 1,304-bp fragment in the parent and a 530-bp fragment in the mutant. Primer pair 2 (FP952/FP1579) amplifies a 566-bp fragment in the parent but no band in the mutant, since primer FP1579 is located in the deleted locus. Lanes L, DNA ladder Gene Ruler 1 kb (GenScript). The positive control was D39 DNA. Lanes C23.1 to C23.3 contain colonies tested after growth for several generations to allow segregation. Download FIG S6, TIF file, 1.7 MB. Copyright © 2017 Junges et al. 2017 Junges et al. This content is distributed under the terms of the Creative Commons Attribution 4.0 International license.

10.1128/mSphere.00324-17.8 TABLE S2  Protocol for C+Y_YB_ medium preparation. Download TABLE S2, PDF file, 0.03 MB. Copyright © 2017 Junges et al. 2017 Junges et al. This content is distributed under the terms of the Creative Commons Attribution 4.0 International license.

10.1128/mSphere.00324-17.9 TABLE S3  Protocol for high-efficiency transformation of *S. pneumoniae* D39. Download TABLE S3, DOCX file, 0.02 MB. Copyright © 2017 Junges et al. 2017 Junges et al. This content is distributed under the terms of the Creative Commons Attribution 4.0 International license.

### Measurement of pneumococcal capsule thickness.

Degree of encapsulation was determined by measuring the zone of exclusion of FITC-dextran (2,000 kDa; Sigma) by the method of Gates et al. (31), and wet mounts were prepared in accordance with reference 29. The preparations were visualized with a Nikon Eclipse CI-L/S microscope (Nikon) with a 100× objective and photographed with a Zyla 5.5 complementary metal oxide semiconductor camera (Andor). The images were analyzed with NIS-Elements BR 3.0 software (Nikon). For each strain, the mean area of FITC exclusion of 200 cells was determined; long chains and clumps of cells were not included.

### Biofilm assay.

We used a protocol for biofilm assays previously described (71). Briefly, a lung cell line (A549; Sigma-Aldrich) was grown to 100% confluence on 24-well plates. At the point of full confluence, the cells were fixed with 4% paraformaldehyde for 30 min at room temperature and washed three times with 1× PBS. Bacterial cultures were seeded into a 500-µl volume of CDM++ to an OD_600_ of 0.1 and incubated for 36 h at 34°C in 5% CO_2_. The medium was changed every 12 h. At the end of 36 h, the supernatant was removed, the wells were scraped, and the material obtained was resuspended in THY and plated. CFU were counted after 24 h of incubation at 37°C in 5% CO_2_.

### Animal models.

Animal experiments were performed with female CD1 mice (Charles River, Inc.) 6 to 8 weeks old. For mixed pairwise infections, the same numbers of bacteria from stocks of different strains were mixed and diluted to the appropriate concentration. All samples were plated onto blood agar plates containing gentamicin (5 µg·ml^−1^) for differentiation from other species and CFU calculation.

For the early lung disease model, 5 × 10^6^ CFU of a strain in 50 µl of 1× PBS were inoculated intranasally under isoflurane general anesthesia. At 24 h postinoculation, animals were killed by intraperitoneal pentobarbital injection and the nasal cavity and lungs were each washed with 1 ml of sterile 1× PBS. Samples from the nasal wash and BALF were stored in ice for immediate dilution and plating. To recover the BALF, a small cut in the trachea was made with a scalpel and 1 ml of sterile fresh 1× PBS was inoculated and recovered for plating (72–74).

For the early lung disease mixed-infection model (competitive assay), 2.5 × 10^6^ CFU each of strain D39 and the Over-EXP mutant in 50 µl were inoculated intranasally under isoflurane general anesthesia. The same protocol for killing and sample collection was used. The competitive index (CI) was calculated by dividing the outcome ratio by the inoculum ratio (72). A CI of <1 indicates that the mutant is attenuated compared to D39, and a CI of >1 indicates that D39 is attenuated compared to the mutant (38).

### Ethics statement.

Our experiments were approved by the UCL Biological Services Ethical Committee and the UK Home Office (project license PPL70/6510). Experiments were performed in accordance with United Kingdom national guidelines for animal use and care under UK Home Office license.

### Statistical analysis.

The Human Oral Microbiome Database (75) and GenBank (76) were used to identify and map genes of each strain. Operon prediction was performed with DOOR (25) and the MicrobesOnline database (26). RT-PCR and FITC-dextran exclusion data for two groups were analyzed with Student’s *t* test, while for three or more groups, we used one-way analysis of variance (ANOVA), followed by Tukey’s *post hoc* test. For the *in vitro* and animal models assays, we used Student’s *t* test and one-way ANOVA, followed by the Tukey or Kruskal-Wallis test, respectively. For the mixed-infection model, CIs were compared with the initial ratio of 1.0 (the predicted CI if there is no difference between the two strains tested) by using Student’s *t* test. Significance was set at a *P* value of < 0.05.

### Data availability.

The RNA-Seq raw read data obtained in this study have been deposited with links to BioProject accession no. PRJNA377718 in the NCBI BioProject database.
