# Supplementary figures and images for: A Quorum-Sensing System That Regulates Streptococcus pneumoniae Biofilm Formation and Surface Polysaccharide Production
Source: mSphere. 2017 Sep 13;2(5):e00324-17. doi: 10.1128/mSphere.00324-17 (PMC5597970; doi:10.1128/mSphere.00324-17)

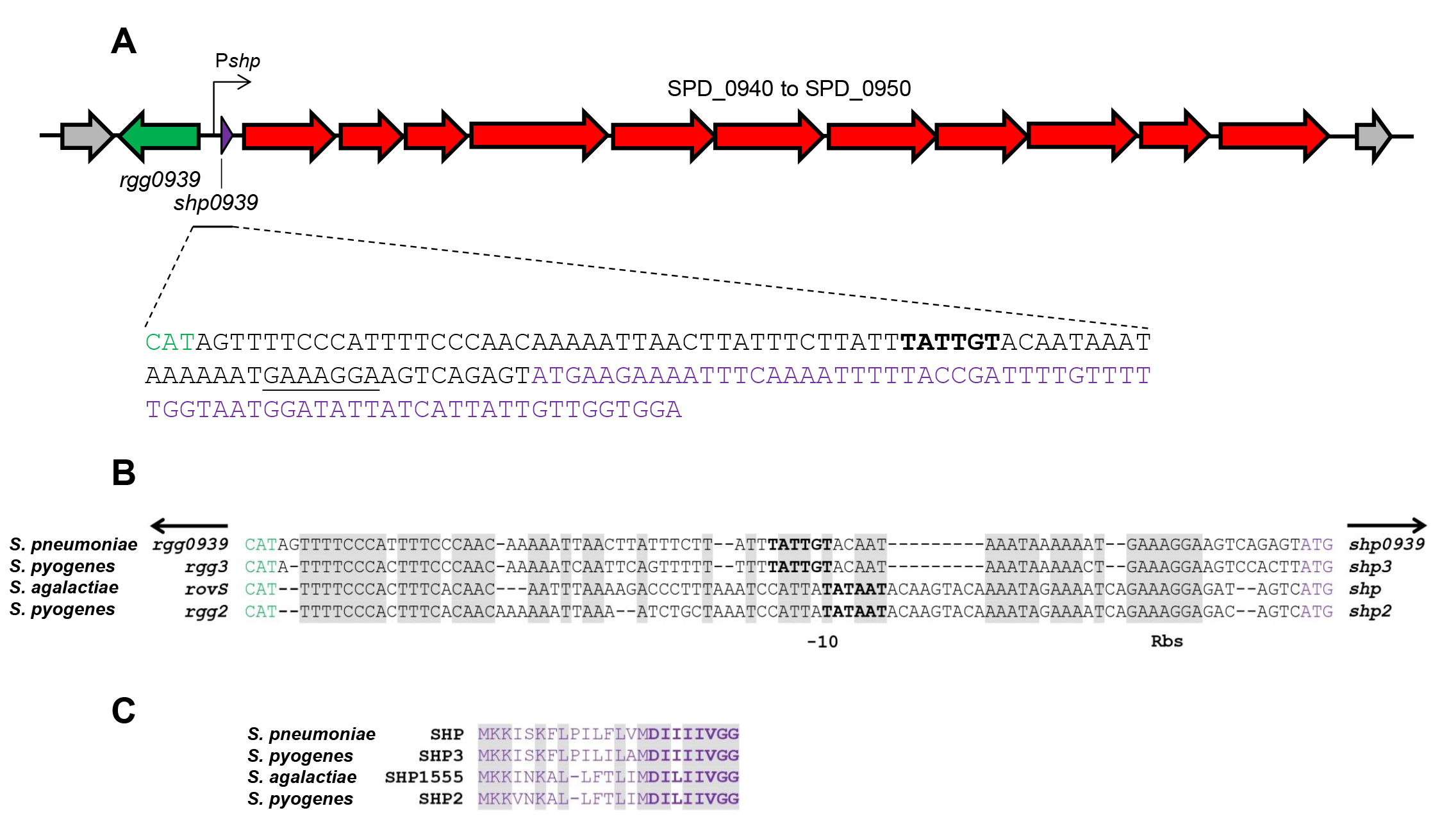

Supplement: FIG S1 [file sph005172355sf1.tif]

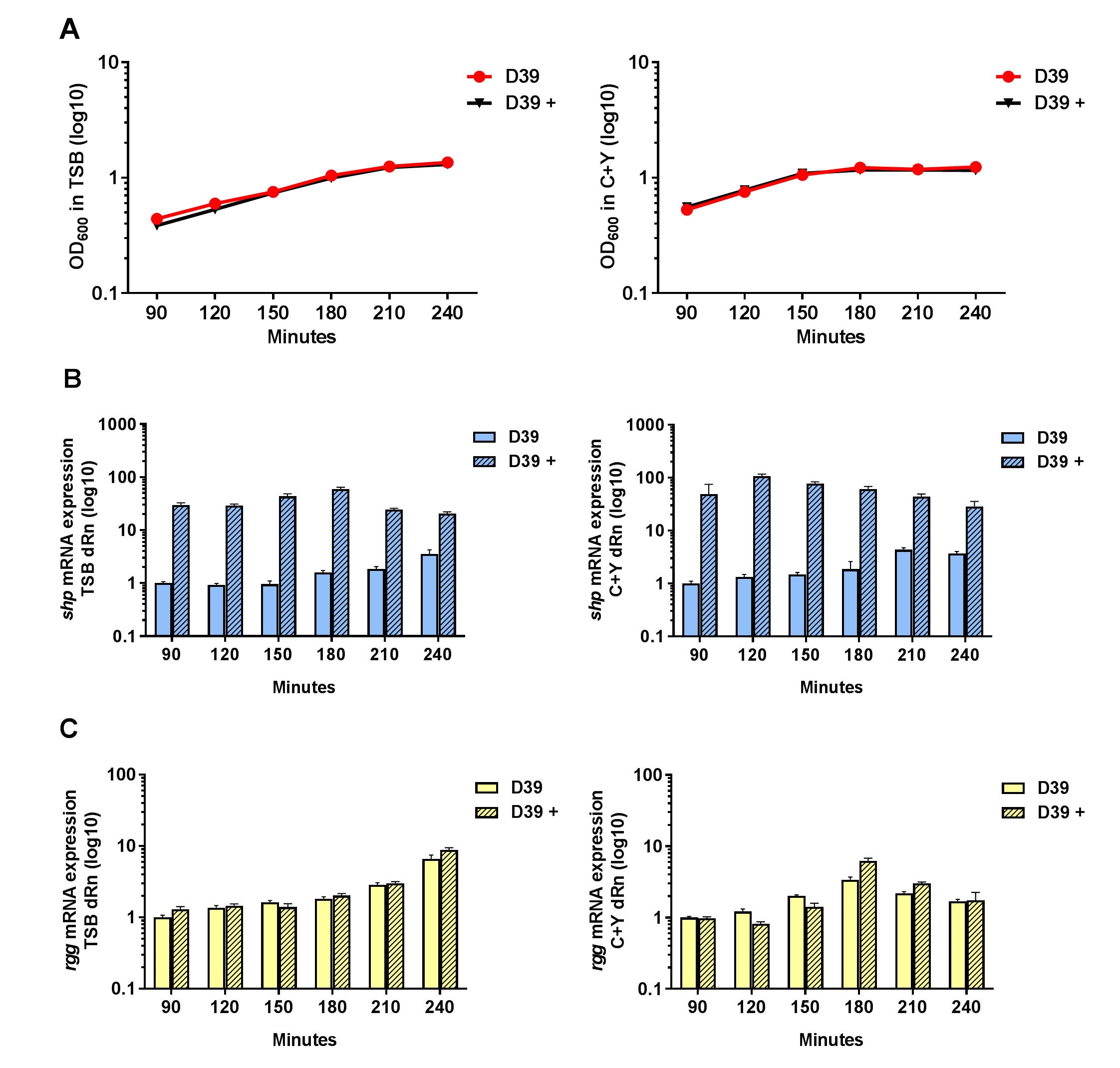

Supplement: FIG S2 [file sph005172355sf2.tif]

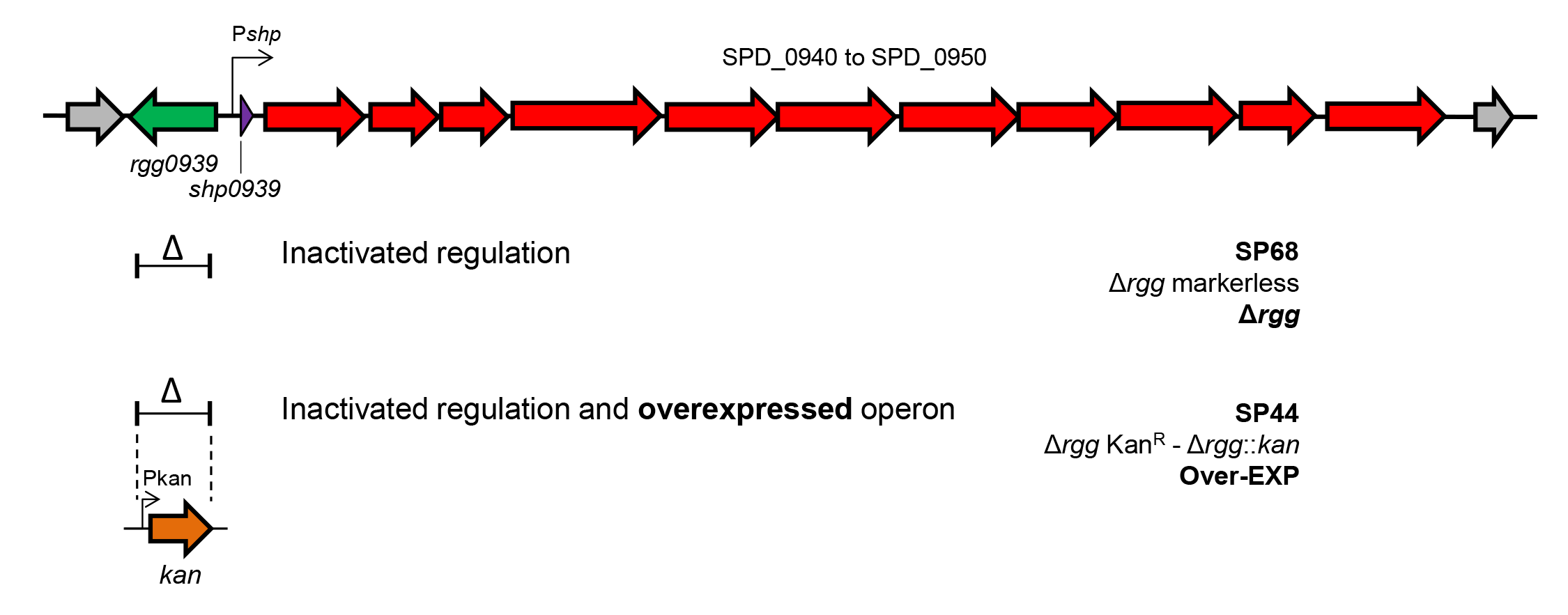

Supplement: FIG S3 [file sph005172355sf3.tif]

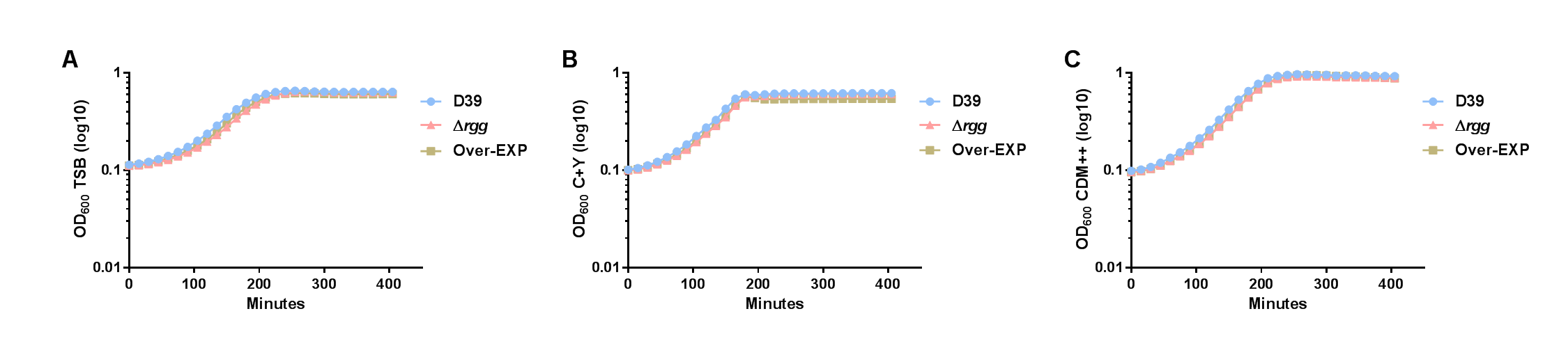

Supplement: FIG S4 [file sph005172355sf4.tif]

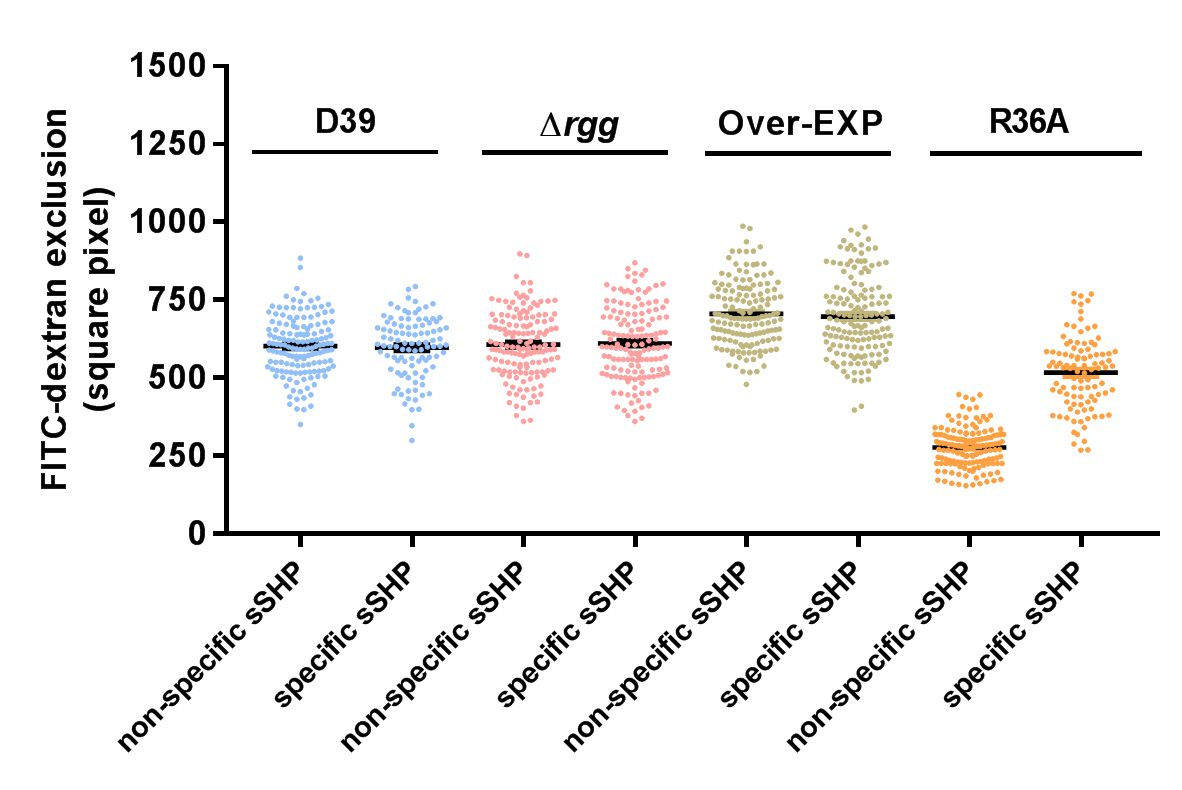

Supplement: FIG S5 [file sph005172355sf5.tif]

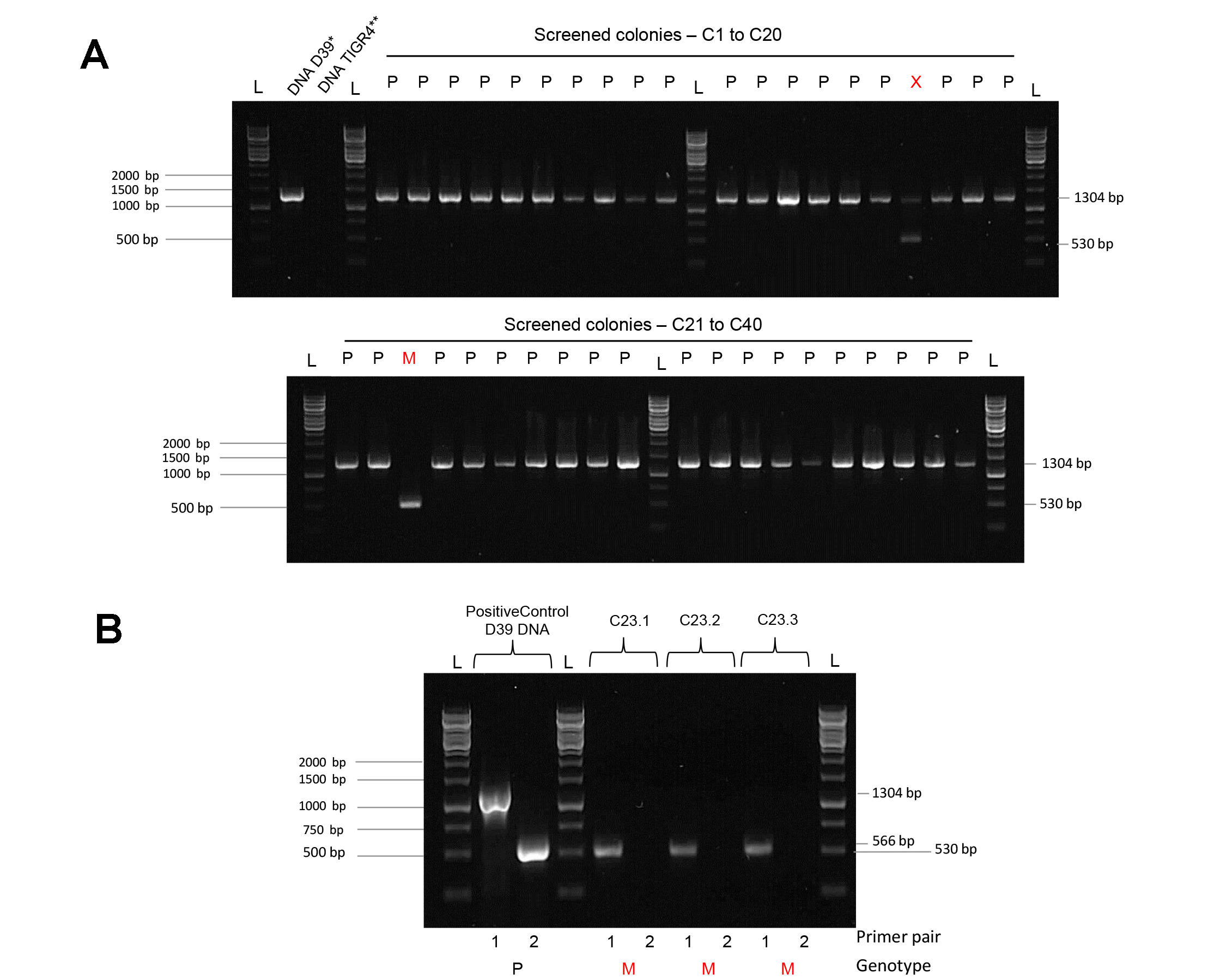

Supplement: FIG S6 [file sph005172355sf6.tif]
