# Supplementary material for: A Quorum-Sensing System That Regulates Streptococcus pneumoniae Biofilm Formation and Surface Polysaccharide Production
Source: mSphere. 2017 Sep 13;2(5):e00324-17. doi: 10.1128/mSphere.00324-17 (PMC5597970; doi:10.1128/mSphere.00324-17)
Supplement: TABLE S1 [file sph005172355st7.docx]

**Table S1.** List of primers used for RT-PCR and construction of mutants.

| **Primers** | **Sequence (5’ to 3’)** | **Gene** | | |
| --- | --- | --- | --- | --- |
| FP367 | ACCGAGGCACGTATGAGCAA | *gyrA* | | |
| FP368 | AGACCAAGGGTTCCCGTTCA |  |  |  |
| FP1014 | ACCGATTTTGTTTTTGGTAATGGA | *shp* | | |
| FP1015 | TGTACACCTAAGCATTTTATCCACCA |  |  |  |
| FP1203 | AGAAAAATTCGTAAGGGCAAACAAA | *rgg0939* | | |
| FP1204 | TTCTGATTCCCCACGTTCAAAA |  |  |  |
| FP1125 | AAATATTACAATTTGGGGTGCAGGA | SPD_0940 | | |
| FP1126 | TTTTGACGCGGAAACCTACCA |  |  |  |
| FP1697 | CATTTAGTTTGGGAACACGAGGAA | SPD_0944 | | |
| FP1698 | ATTCCAACCGCCTTCTCCAAT |  |  |  |
| FP1699 | TGGATGGTCTTGGGGAGTTTC | SPD_0947 | | |
| FP1700 | AAAAGCCCACACTGCAAAAGG |  |  |  |
| FP1701 | TGCCAGTTGGGTCTGGATTTT | SPD_0950 | | |
| FP1702 | CATACCTCTTCAATAGCGTGCTGAAT |  |  |  |
| FP1703 | GTTGGATTTCGGTTGCAGGAG | SPD_0952 | | |
| FP1704 | TGCTGTTTGGAGAATCGGTGA |  |  |  |
| **Primers** | **Sequence (5’ to 3’)** | **Location** | **Template** | **Purpose** |
| FP1573 | GGGGGATAGGAAGGAATTAGC | Downstream  of *rgg0939* | NCTC7466 | Construction of mutant SP068 (fragment 1) |
| FP1603 | AAATTCGTAAGGGCAAACAAATTGACCATTTTAATAAACCAGTAAACAA |  |  |  |
| FP1604 | TTGTTTACTGGTTTATTAAAATGGTCAATTTGTTTGCCCTTACGAATTT | Upstream  of *rgg0939* |  | Construction of mutant SP068 (fragment 2) |
| FP1576 | AAAATTCAAACTTCACATCGTAAAAA |  |  |  |
| FP1577 | GTACGGCTTATTCCGTGAGC | Nested PCR for markerless deletion of *rgg0939* | Fragments 1 and 2 | Create a final amplicon for transformation |
| FP1578 | CCTATCACCCATGGCCTCTA |  |  |  |
| FP952 | AAAATTTCACGAGAATGCCTTA | Flanking regions of *rgg0939* | D39 (parent) and SP068 (mutant) | Screening of SP068 |
| FP1126 | TTTTGACGCGGAAACCTACCA |  |  |  |
| FP1579 | TGGATTCGAACATCATTCCAT |  |  |  |
| FP820 | CGTTCGTGAATTTGAAGTGG | Upstream  of *rgg0939* | NCTC7466 | Construction of mutant SP044 (fragment 1) |
| FP949 | AGGCGCGCCCCATACAAGTCTTCGATATTTTAGGA |  |  |  |
| FP950 | AGGCCGGCCTGATTCCCCACGTTCAAA | Downstream  of *rgg0939* |  | Construction of mutant SP044 (fragment 2) |
| FP951 | CCAATGTACCAATAACTCGAGGA |  |  |  |
| FP948 | CAAGGTGTCTTTCGGTGTCA | Nested PCR for deletion of *rgg0939* | Fragments 1 and 2 | Create a final amplicon for transformation |
| FP964 | TGCAAATCATTCAACTTTGGTC |  |  |  |
| FP001 | AGGCGCGCCGTTTGATTTTTAATG | Kan^R^ gene | pSF151 | Kan marker |
| FP068 | AGGCCGGCCTAGGTACTAAAACAATTCATCCAGTA |  |  |  |

*Underlined letters represent restriction sites; AscI – AGGCGCGCC; FseI – AGGCCGGCC.
