# Supplementary material for: A Quorum-Sensing System That Regulates Streptococcus pneumoniae Biofilm Formation and Surface Polysaccharide Production
Source: mSphere. 2017 Sep 13;2(5):e00324-17. doi: 10.1128/mSphere.00324-17 (PMC5597970; doi:10.1128/mSphere.00324-17)
Supplement: TABLE S2 [file sph005172355st8.pdf]

# Table S2. Protocol for C+$Y_{YB}$ medium preparation.

In this protocol we describe how we prepare 1L of C+$Y_{YB}$ medium.

**1.** Add the following components to a sterile flask in this order:

| Media component | Concentration |
|---|---|
| **arginine** | 150 mg/L |
| **asparagine** | 40 mg/L |
| **cystine** | 120 mg/L |
| **glutamine** | 20 mg/L |
| **glycine** | 80 mg/L |
| **isoleucine** | 200 mg/L |
| **leucine** | 400 mg/L |
| **serine** | 250 mg/L |
| **valine** | 250 mg/L |
| **$CaCl_2$** | 2.5 mg/L |
| **$MgCl_2 \cdot 6H_2O$** | 0.5 g/L |
| **maltose** | 2 g/L |
| **adenine** | 5 mg/L |
| **sodium acetate** | 2 g/L |
| **sodium pyruvate** | 240 mg/L |
| **nicotinic acid** | 0.2 mg/L |
| **pyridoxine hydrochloride** | 0.2 mg/L |
| **D-calcium pantothenate** | 0.6 mg/L |
| **thiamine hydrochloride** | 0.2 mg/L |
| **riboflavin** | 0.1 mg/L |
| **choline chloride** | 4 mg/L |
| **yeast exract** | 8 g/L |
| **bovine serum albumin** | 0.48 g/L |
| **NaCl** | 2 g/L |

**2.** Add 1L of sterile distilled water and mix by shaking.

**3.** Add the liquid components to give these concentrations:

| | |
|---|---|
| **potassium phosphate buffer, pH 7.4** | 50 mM |
| **- 1M solution - $K_2HPO_4$** | 11.2 mL |
| **- 1M solution - $KH_2PO_4$** | 38.8 mL |
| **$MnSO_4 \cdot 4H_2O$** | 25 µg/L* |
| **biotin** | 0.2 µg/L* |

* $MnSO_4 \cdot 4H_2O$ stock preparation: 1mL distilled water + 0.0025g $MnSO_4 \cdot 4H_2O$. <stable at 4C>

- Add 10µL of the stock to 1L of medium (final concentration of 25 µg/L)

* Biotin stock preparation: 10mL distilled water + 0.0002g biotin. <stable at 4C>

- Add 10µL of the stock to 1L medium (final concentration of 0.2 µg/L)

**4.** Mix the medium in a magnetic stirrer for 30-60 minutes.

**5.** Filter the medium through 0.2 µm filter.

**6.** Store at 4C and protected from light.
