# Supplementary material for: A Quorum-Sensing System That Regulates Streptococcus pneumoniae Biofilm Formation and Surface Polysaccharide Production
Source: mSphere. 2017 Sep 13;2(5):e00324-17. doi: 10.1128/mSphere.00324-17 (PMC5597970; doi:10.1128/mSphere.00324-17)
Supplement: TABLE S3 [file sph005172355st9.docx]

**Table S3. Protocol for high efficiency transformation of *S. pneumoniae* D39.**

1. Prepare pre-culture stocks of D39 at OD_600_ 0.5 in TSB.
2. Dilute 100µL of the pre-culture 1:100 in C+Y_YB_.
3. Incubate at 37C 5%CO_2_ until it reaches OD_600_ 0.04 (~2 hours).
4. Transfer 500µL of the culture to a microtube. Add CSP-1 to a final concentration of 50nM and add donor DNA*. Mix by pipetting up and down.
5. Incubate at 37C for 3 hours.
6. After the incubation period, prepare serial dilutions of the culture down to 10^-7^. If the mutation that is being inserted carries a marker, plate a few dilutions in selective plates for mutant recovery. If we expect good efficiency, we often plate down to 10^-4^ to ensure that we can isolate a colony. In addition, if desired, plate down to the 10^-6^ or 10^-7^ dilution in plain plates for calculation of efficiency, which should vary according to the donor DNA** used.

* For genomic DNA, we use about 5µg/mL, and for PCR amplicons with 2-3kb flanking regions, we use 200ng/mL.

** Better efficiency will be observed when using donor PCR amplicon with 2-3kb flanking regions compared to genomic DNA
